# Peripapillary retinal nerve fiber layer thickness in patients with unilateral retinal vein occlusion

**DOI:** 10.1038/s41598-021-97693-7

**Published:** 2021-09-13

**Authors:** Jayoung Ahn, Daniel Duck-Jin Hwang

**Affiliations:** 1 Department of Ophthalmology, Hangil Eye Hospital, 35 Bupyeong-daero, Bupyeong-gu, Incheon, 21388 Korea; 2 Department of Ophthalmology, Catholic Kwandong University College of Medicine, Incheon, Korea

**Keywords:** Optic nerve diseases, Retinal diseases

## Abstract

This study evaluated longitudinal changes in peripapillary retinal nerve fiber layer (pRNFL) thickness in eyes affected with branch and central retinal vein occlusion (BRVO and CRVO, respectively) and fellow eyes. This retrospective case–control study included patients with newly diagnosed unilateral BRVO (46 patients) or unilateral CRVO (27 patients). The control group included 48 patients without abnormal findings on the fundus examination. Global and all-sector pRNFL thicknesses were greater in eyes with BRVO and CRVO than in fellow eyes at baseline; however, at 24 months, this difference remained only in the temporal sector of eyes affected with CRVO. Although the global pRNFL thicknesses of the fellow eyes in the BRVO and CRVO groups decreased significantly at 24 months compared to baseline (p = 0.001 and p = 0.011, respectively), there was no significant difference in the normal control group (p = 0.824). The global, inferior temporal, and inferior nasal pRNFL thicknesses at 12 and 24 months were significantly lower in the fellow eyes of the CRVO group than in those of the BRVO and normal control groups. The fellow eyes of patients with BRVO and CRVO suffered a significant reduction in pRNFL thickness compared to normal controls, indicating that they are susceptible to pRNFL damage.

## Introduction

Retinal vein obstruction (RVO) is the second most common disease following diabetic retinopathy among retinal vascular diseases that cause vision loss^1^. RVO is classified as branch retinal vein occlusion (BRVO) or central retinal vein occlusion (CRVO) according to the location of the occluded vein^2^, with BRVO being four to six times more common than CRVO^3–6^.

The prevalence of glaucoma is higher in patients with RVO than in the general population^7,8^. The mean intraocular pressure (IOP) is reportedly higher in patients with RVO than in normal controls, and it has been hypothesized that elevated IOP results in blood vessel compression and subsequent intimal proliferation, leading to RVO^9,10^. Additionally, pathophysiological vascular abnormalities caused by systemic diseases such as hypertension and diabetes induce both glaucomatous optic neuropathy and RVO^11–16^, suggesting that insulin resistance and autoregulatory dysfunction may be the causes of both diseases^17^.

To confirm the association between RVO and glaucoma, several previous studies have investigated the peripapillary retinal nerve fiber layer (pRNFL)^14,18–22^. It has been reported that, two years after unilateral BRVO diagnosis, the pRNFL thickness in the affected eye is thinner than that of the normal fellow eye^19^. In a study that aimed to confirm the hypothesis that RVO and glaucoma share systemic vascular abnormalities, the pRFNL thickness was thinner in the normal fellow eyes of patients with unilateral RVO than in those of normal controls^14,20,21^.

However, previous studies have analyzed either BRVO and CRVO patients together^14,20,22^ or exclusively BRVO patients^18,19,21^. Kim et al.^14^ classified RVO according to the location of the occluded vein as arteriovenous crossing RVO (AV-RVO), optic nerve site RVO (ON-RVO), or optic nerve site RVO with optic nerve swelling (ONHS-RVO), to subsequently perform a subgroup analysis; they found no difference in the pRNFL thickness between the AV-RVO and ON-RVO groups. However, given that this was a cross-sectional study and, therefore, focused solely on the moment of diagnosis, follow-up changes were not included. Furthermore, patients with ONHS-RVO (five eyes) were excluded from analysis because of the small sample size.

Longitudinal comparisons of pRNFL thickness in BRVO and CRVO have not been reported to date, and the difference in the degree of glaucomatous damage between the two diseases is unknown. Therefore, this study aimed to compare longitudinal changes in pRNFL thickness between the affected and fellow eyes of patients with unilateral BRVO and unilateral CRVO.

## Results

We included 92 eyes of 46 patients with unilateral BRVO, 54 eyes of 27 patients with unilateral CRVO, and 48 eyes of 48 normal controls. Based on the horizontal raphe, 30 eyes had superior BRVO, and 16 eyes had inferior BRVO. There were no significant differences in baseline characteristics among the three groups (Table 1).

**Table 1:** Baseline patient characteristics.

	BRVO	CRVO	Controls	p-value
Patients	46	27	48	
Age (years)	63.20 ± 10.02	65.19 ± 15.11	63.44 ± 10.31	0.753^a^
**Sex**				0.216^b^
Male	14	12	12	
Female	32	15	36	
**Affected eye**				0.384^b^
OD	24	10	25	
OS	22	17	23	
**Systemic disease**				
Hypertension	21	15	25	0.685^b^
Diabetes	9	9	8	0.222^b^

Values are presented as the number or mean ± standard deviation.

*BRVO* branch retinal vein occlusion, *CRVO* central retinal vein occlusion, *OD* right eye, *OS* left eye.

^a^p-value derived from a one-way analysis of variance.

^b^p-value derived from Pearson’s chi-square test.

During the study period, 2.46 ± 2.20 intravitreal anti-vascular endothelial growth factor injections were administered to the affected eyes of patients with BRVO, and 3.74 ± 3.78 injections were administered to the affected eyes of patients with CRVO. The number of injections was not significantly different between the BRVO and CRVO groups (p = 0.115, derived from independent samples *t*-test). Scatter laser photocoagulation was performed in 5 of the affected eyes of patients with BRVO. In patients with CRVO, pan-retinal laser photocoagulation was performed in 9 of the affected eyes, and scatter laser photocoagulation was performed in 1 eye.

### pRNFL thickness in the BRVO group

The global and all-sector pRNFL thicknesses of the affected eyes decreased significantly at 24 months compared to baseline (Table 2). In the fellow eyes, only the global pRNFL thickness decreased significantly at 24 months (p = 0.001).

**Table 2:** Comparison of peripapillary retinal nerve fiber layer thickness (μm) in the unilateral branch retinal vein occlusion group.

	Affected eye	Fellow eye	p-value^a^
**Global pRNFL**			
Baseline	119.15 ± 17.71	104.52 ± 10.46	< 0.001*
6th month	105.76 ± 11.54	104.07 ± 10.58	0.179
12th month	103.70 ± 12.33	103.87 ± 10.33	0.899
24th month	101.60 ± 13.32	103.35 ± 10.21	0.159
p-value^b^	< 0.001*	0.001*	
**Superior temporal sector pRNFL**			
Baseline	161.28 ± 31.33	144.35 ± 22.64	0.001*
6th month	140.84 ± 30.26	145.13 ± 21.71	0.359
12th month	135.63 ± 32.48	145.04 ± 21.67	0.048*
24th month	130.19 ± 34.54	143.74 ± 21.11	0.007*
p-value^b^	< 0.001*	0.546	
**Temporal sector pRNFL**			
Baseline	101.15 ± 31.00	76.85 ± 12.85	< 0.001*
6th month	82.53 ± 14.69	75.87 ± 12.32	0.002*
12th month	81.37 ± 16.24	75.78 ± 11.86	0.013*
24th month	78.71 ± 11.83	75.67 ± 11.75	0.103
p-value^b^	< 0.001*	0.084	
**Inferior temporal sector pRNFL**			
Baseline	167.30 ± 40.40	151.26 ± 19.36	0.004*
6th month	149.71 ± 32.00	152.29 ± 17.25	0.564
12th month	148.22 ± 31.46	151.93 ± 17.34	0.449
24th month	143.24 ± 36.12	152.00 ± 17.86	0.099
p-value^b^	0.002*	0.606	
**Inferior nasal sector pRNFL**			
Baseline	126.15 ± 29.88	117.02 ± 18.83	0.036*
6th month	115.36 ± 20.87	115.47 ± 20.03	0.968
12th month	112.98 ± 21.92	115.07 ± 19.13	0.442
24th month	111.50 ± 20.96	114.80 ± 20.28	0.267
p-value^b^	0.004*	0.172	
**Nasal sector pRNFL**			
Baseline	81.70 ± 17.43	74.20 ± 11.73	0.004*
6th month	76.11 ± 12.14	73.73 ± 12.17	0.158
12th month	74.50 ± 12.08	73.11 ± 11.58	0.382
24th month	74.90 ± 13.39	73.20 ± 11.63	0.549
p-value^b^	0.015*	0.076	
**Superior nasal sector pRNFL**			
Baseline	132.80 ± 33.37	120.35 ± 25.96	0.011*
6th month	122.53 ± 26.98	120.42 ± 22.08	0.572
12th month	120.57 ± 27.26	120.22 ± 21.53	0.925
24th month	119.83 ± 27.73	118.93 ± 20.41	0.622
p-value^b^	< 0.001*	0.561	

^a^Comparison between the affected and fellow eyes during each period (paired *t-*tests).

^b^Comparison between the baseline and 24-month values (paired *t*-tests).

Asterisks (*) indicate statistically significant p-values.

*pRNFL* peripapillary retinal nerve fiber layer.

At baseline, the global and all-sector pRNFL thicknesses were significantly greater in the affected eyes than in the fellow eyes. The temporal sector pRNFL thickness of the affected eyes was greater than that of the fellow eyes at 6 and 12 months (p = 0.002 and p = 0.013, respectively). In contrast, the superior temporal pRNFL thickness was significantly lower in the affected eyes at 12 and 24 months (p = 0.048 and p = 0.007, respectively).

### pRNFL thickness in the CRVO group

The global and all-sector pRNFL thicknesses of the affected eyes decreased significantly at 24 months compared to the baseline (Table 3). Fellow eyes showed a significant decrease in global, superior temporal sector, and inferior temporal sector pRNFL thicknesses at 24 months (p = 0.011, p = 0.003, and p = 0.022, respectively).

**Table 3:** Comparison of peripapillary retinal nerve fiber layer thickness (μm) in the unilateral central retinal vein occlusion group.

	Affected eye	Fellow eye	p-value^a^
**Global pRNFL**			
Baseline	136.04 ± 36.33	99.93 ± 13.59	< 0.001*
6th month	118.46 ± 34.12	98.81 ± 14.05	0.005*
12th month	106.50 ± 18.95	97.00 ± 14.44	0.001*
24th month	101.48 ± 25.85	96.36 ± 14.61	0.254
p-value^b^	< 0.001*	0.011*	
**Superior temporal sector pRNFL**			
Baseline	168.96 ± 40.85	138.85 ± 22.74	< 0.001*
6th month	154.13 ± 43.15	136.88 ± 25.88	0.087
12th month	143.58 ± 33.68	133.70 ± 24.75	0.299
24th month	129.08 ± 37.14	131.12 ± 26.39	0.804
p-value^b^	0.001*	0.003*	
**Temporal sector pRNFL**			
Baseline	127.11 ± 54.33	76.26 ± 14.28	< 0.001*
6th month	101.08 ± 43.76	76.81 ± 14.24	0.011*
12th month	90.38 ± 24.55	76.13 ± 14.73	0.003*
24th month	87.84 ± 31.98	73.60 ± 15.02	0.006*
p-value^b^	0.002*	0.361	
**Inferior temporal sector pRNFL**			
Baseline	168.70 ± 53.62	143.41 ± 28.56	0.012*
6th month	163.29 ± 38.39	141.77 ± 26.09	0.002*
12th month	146.29 ± 37.98	135.87 ± 24.52	0.201
24th month	136.60 ± 36.58	134.92 ± 24.70	0.774
p-value^b^	0.017*	0.022*	
**Inferior nasal sector pRNFL**			
Baseline	147.59 ± 54.64	106.48 ± 27.59	0.001*
6th month	128.29 ± 40.81	105.58 ± 24.20	0.025*
12th month	113.75 ± 29.41	99.35 ± 24.38	0.053
24th month	104.32 ± 21.87	102.48 ± 24.72	0.758
p-value^b^	0.003*	0.094	
**Nasal sector pRNFL**			
Baseline	100.96 ± 43.76	71.78 ± 16.69	0.001*
6th month	87.63 ± 35.93	69.81 ± 18.47	0.026*
12th month	74.67 ± 20.43	68.61 ± 17.91	0.134
24th month	74.28 ± 25.60	69.24 ± 17.29	0.315
p-value^b^	0.016*	0.541	
**Superior nasal sector pRNFL**			
Baseline	146.07 ± 48.54	114.59 ± 26.45	0.002*
6th month	125.29 ± 54.52	112.27 ± 26.19	0.244
12th month	117.58 ± 33.39	109.87 ± 27.24	0.256
24th month	112.16 ± 38.65	110.52 ± 25.23	0.804
p-value^b^	0.003*	0.115	

^a^Comparison between affected and fellow eyes in each period (paired *t*-test).

^b^Comparison between the baseline and 24-month values (paired *t-*test).

Asterisks (*) indicate statistically significant p-values.

*pRNFL* peripapillary retinal nerve fiber layer.

At baseline, the global and all-sector pRNFL thicknesses were significantly greater in the affected eyes than in the fellow eyes. The global pRNFL thickness was greater in the affected eyes than in the fellow eyes by 12 months. The temporal sector pRNFL thickness was greater in the affected eyes than in the fellow eyes throughout the entire 24-month study period. The inferior temporal sector, inferior nasal sector, and nasal sector pRNFL thicknesses were significantly greater in the affected eyes than in the fellow eyes at 6 months (p = 0.002, p = 0.025, and p = 0.026, respectively).

### Comparison of pRNFL thickness between the fellow eyes of patients with BRVO, CRVO, and normal controls

In the normal controls, there were no significant differences in global or all-sector pRNFL thicknesses at 24 months compared to baseline (Table 4). Additionally, there were no differences among the three groups regarding pRNFL thickness in any sector at baseline.

**Table 4:** Comparison of peripapillary retinal nerve fiber layer thickness (μm) between fellow eyes and normal control eyes.

	BRVO group	CRVO group	p-value^a^	Control	p-value^b^	p-value^c^
**G pRNFL**						
Baseline	104.52 ± 10.46	99.93 ± 13.59	0.110	103.40 ± 10.54	0.605	0.222
6th month	104.07 ± 10.58	98.81 ± 14.05	0.079	103.39 ± 9.61	0.765	0.126
12th month	103.87 ± 10.33	97.00 ± 14.44	0.026*	103.29 ± 10.52	0.789	0.041*
24th month	103.35 ± 10.21	96.36 ± 14.61	0.021*	103.25 ± 10.62	0.964	0.024*
p-value^d^	0.001*	0.011*		0.824		
**ST pRNFL**						
Baseline	144.35 ± 22.64	138.85 ± 22.74	0.321	143.75 ± 18.95	0.890	0.321
6th month	145.13 ± 21.71	136.88 ± 25.88	0.155	145.84 ± 14.53	0.860	0.118
12th month	145.04 ± 21.67	133.70 ± 24.75	0.055	142.63 ± 19.81	0.573	0.106
24th month	143.74 ± 21.11	131.12 ± 26.39	0.031*	144.00 ± 19.94	0.951	0.022*
p-value^d^	0.546	0.003*		0.848		
**T pRNFL**						
Baseline	76.85 ± 12.85	76.26 ± 14.28	0.857	78.38 ± 11.58	0.546	0.488
6th month	75.87 ± 12.32	76.81 ± 14.24	0.771	77.50 ± 10.08	0.516	0.820
12th month	75.78 ± 11.86	76.13 ± 14.73	0.916	79.10 ± 11.70	0.175	0.361
24th month	75.67 ± 11.75	73.60 ± 15.02	0.522	79.08 ± 11.03	0.150	0.080
p-value^d^	0.084	0.361		0.125		
**IT pRNFL**						
Baseline	151.26 ± 19.36	143.41 ± 28.56	0.166	147.06 ± 27.65	0.398	0.589
6th month	152.29 ± 17.25	141.77 ± 26.09	0.074	147.95 ± 19.27	0.282	0.280
12th month	151.93 ± 17.34	135.87 ± 24.52	0.002*	150.27 ± 20.47	0.672	0.011*
24th month	152.00 ± 17.86	134.92 ± 24.70	0.004*	150.25 ± 19.37	0.650	0.005*
p-value^d^	0.606	0.022*		0.206		
**IN pRNFL**						
Baseline	117.02 ± 18.83	106.48 ± 27.59	0.086	114.25 ± 21.32	0.506	0.178
6th month	115.47 ± 20.03	105.58 ± 24.20	0.068	116.55 ± 21.71	0.813	0.063
12th month	115.07 ± 19.13	99.35 ± 24.38	0.005*	114.23 ± 21.15	0.841	0.010*
24th month	114.80 ± 20.28	102.48 ± 24.72	0.027*	114.23 ± 21.27	0.894	0.038*
p-value^d^	0.172	0.094		0.740		
**N pRNFL**						
Baseline	74.20 ± 11.73	71.78 ± 16.69	0.511	70.40 ± 11.28	0.113	0.703
6th month	73.73 ± 12.17	69.81 ± 18.47	0.284	71.42 ± 11.38	0.377	0.694
12th month	73.11 ± 11.58	68.61 ± 17.91	0.281	71.23 ± 11.52	0.432	0.526
24th month	73.20 ± 11.63	69.24 ± 17.29	0.255	70.37 ± 11.93	0.249	0.743
p-value^d^	0.076	0.541		0.788		
**SN pRNFL**						
Baseline	120.35 ± 25.96	114.59 ± 26.45	0.367	121.33 ± 24.15	0.849	0.266
6th month	120.42 ± 22.08	112.27 ± 26.19	0.166	119.82 ± 22.61	0.902	0.223
12th month	120.22 ± 21.53	109.87 ± 27.24	0.090	119.33 ± 22.84	0.847	0.130
24th month	118.93 ± 20.41	110.52 ± 25.23	0.132	119.06 ± 23.89	0.978	0.159
p-value^d^	0.561	0.115		0.054		

^a^Comparison between the BRVO fellow eyes and CRVO fellow eyes in each period (independent samples *t*-test).

^b^Comparison between the BRVO fellow eyes and normal control eyes (independent samples *t*-test).

^c^Comparison between CRVO fellow eyes and normal control eyes (independent samples *t*-test).

^d^Comparison between the baseline and 24-month values (paired *t*-test).

Asterisks (*) indicate statistically significant p-values.

*pRNFL* peripapillary retinal nerve fiber layer, *G* global, *ST* superior temporal, *T* temporal, *IT* inferior temporal, *IN* inferior nasal, *N* nasal, *SN* superior nasal.

There were no significant differences in the global or all-sector pRNFL thicknesses of the normal controls compared to the fellow eyes of patients with BRVO at any of the visits. However, compared with normal controls, pRNFL thicknesses in the global, inferior temporal, and inferior nasal sectors at 12 and 24 months were significantly lower in the fellow eyes of patients with CRVO. When comparing fellow eyes in BRVO and CRVO, the latter presented with a lower pRNFL thickness in the global, inferior temporal, and inferior nasal sectors at 12 and 24 months.

**Figure 1:**
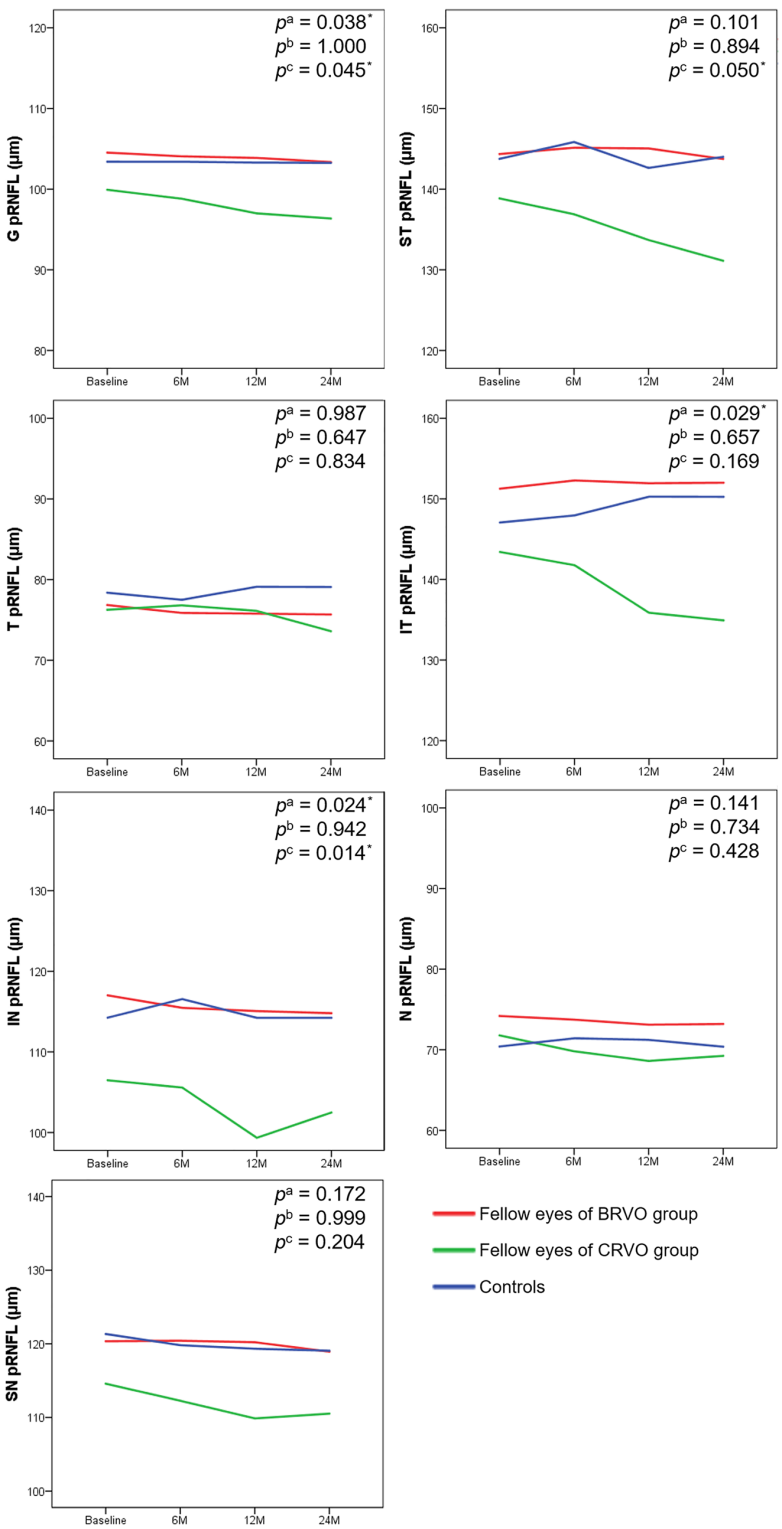
Changes in the peripapillary retinal nerve fiber layer thickness (pRNFL) in fellow eyes and in normal control eyes. The p-values (derived from a repeated measures analysis of variance) are shown for the differences between groups regarding the amount of pRNFL change over time. p^a^ Comparison between fellow BRVO and fellow CRVO eyes. p^b^ Comparison between fellow BRVO eyes and normal controls. p^c^ Comparison between fellow CRVO and normal control eyes. *G* global, *ST* superior temporal, *T* temporal, *IT* inferior temporal, *IN* inferior nasal, *N* nasal, *SN* superior nasal.

The changes in global, superior temporal sector, and inferior nasal sector pRNFL thicknesses over time were significantly greater in the fellow eyes of patients with CRVO than in normal controls (p = 0.045, p = 0.050, and p = 0.014, respectively) (Fig. 1). Additionally, the changes in global, inferior temporal sector, and inferior nasal sector pRNFL thicknesses were significantly different in the fellow eyes of patients with CRVO compared with the fellow eyes of patients with BRVO (p = 0.038, p = 0.029, and p = 0.024, respectively).

### Best-corrected visual acuity, IOP, and central macular thickness variances

In the BRVO group, the best-corrected visual acuity (BCVA) of the affected eyes had improved significantly at 24 months compared to baseline, whereas in the fellow eyes it showed no significant change (Table 5). The BCVA of the affected eyes was lower than that of the fellow eyes at all visits. In the CRVO group, no significant change was observed in BCVA in either the affected or the fellow eyes at 24 months compared to baseline. However, at all visits, the BCVA of the affected eyes was lower than that of the fellow eyes.

**Table 5:** Best-corrected visual acuity, intraocular pressure, and central macular thickness variances in eyes with retinal vein occlusion.

	Affected eye	Fellow eye	p-value^a^
**BRVO group**			
BCVA (logMAR)			
Baseline	0.46 ± 0.43	0.04 ± 0.08	< 0.001*
6th month	0.23 ± 0.32	0.04 ± 0.08	< 0.001*
12th month	0.24 ± 0.32	0.05 ± 0.08	< 0.001*
24th month	0.23 ± 0.29	0.05 ± 0.07	< 0.001*
p-value^b^	0.001*	0.431	
**IOP (mmHg)**			
Baseline	14.98 ± 2.93	15.52 ± 2.92	0.091
6th month	14.96 ± 3.99	15.20 ± 2.96	0.603
12th month	14.63 ± 3.04	14.29 ± 2.99	0.253
24th month	14.66 ± 3.21	14.78 ± 3.38	0.904
p-value^b^	0.487	0.088	
**CMT(μm)**			
Baseline	468.33 ± 194.17	262.59 ± 26.52	< 0.001*
6th month	325.93 ± 130.32	265.15 ± 26.26	0.004*
12th month	296.30 ± 80.92	265.11 ± 28.87	0.017*
24th month	298.45 ± 75.54	265.89 ± 27.17	0.010*
p-value^b^	< 0.001*	0.339	
CRVO group			
**BCVA (logMAR)**			
Baseline	0.72 ± 0.72	0.13 ± 0.19	< 0.001*
6th month	0.57 ± 0.60	0.12 ± 0.17	0.001*
12th month	0.54 ± 0.59	0.12 ± 0.14	0.002*
24th month	0.66 ± 0.71	0.13 ± 0.17	0.001*
p-value^b^	0.517	0.937	
**IOP (mmHg)**			
Baseline	15.74 ± 2.98	15.85 ± 3.02	0.855
6th month	16.15 ± 3.25	16.20 ± 2.77	0.864
12th month	16.50 ± 3.48	15.84 ± 3.42	0.270
24th month	15.62 ± 3.48	15.52 ± 3.00	0.832
p-value^b^	0.881	0.185	
**CMT(μm)**			
Baseline	563.22 ± 247.01	264.22 ± 29.47	< 0.001*
6th month	393.92 ± 195.86	262.78 ± 24.09	0.003*
12th month	415.81 ± 212.94	267.48 ± 25.82	0.002*
24th month	356.96 ± 134.09	263.96 ± 28.31	0.001*
p-value^b^	0.001*	1.000	

^a^Comparison between the affected and fellow eyes in each period (paired *t*-test).

^b^Comparison between the baseline and 24-month values (paired *t*-test).

Asterisks (*) indicate statistically significant p-values.

*BRVO* branch retinal vein occlusion, *CRVO* central retinal vein occlusion, *BCVA* best-corrected visual acuity, *logMAR* logarithm of the minimum angle of resolution, *IOP* intraocular pressure.

In both RVO groups, there was no significant difference in IOP in either the affected or the fellow eyes at 24 months compared to baseline. Additionally, no significant differences in IOP were observed between the affected and fellow eyes at any of the visits.

In both groups, the central macular thickness (CMT) of the affected eyes decreased significantly at 24 months compared to baseline (BRVO group, p < 0.001; CRVO group, p = 0.001); no difference was observed in the fellow eyes. In both groups, the CMT of the affected eyes was greater than that of the fellow eyes at all visits.

## Discussion

To the best of our knowledge, this is the first study to compare longitudinal changes in pRNFL thickness between patients with BRVO and CRVO. We found that global and all-sector pRNFL thicknesses were greater in the affected eyes than in the fellow eyes of patients with these conditions at baseline; however, at 24 months, only the temporal sector of the affected eyes of patients with CRVO remained significantly thicker. Furthermore, the global pRNFL thickness of the fellow eyes decreased significantly at 24 months compared to baseline in the BRVO and CRVO groups, while remaining stable in the normal control group. In addition, we found that the fellow eyes of the CRVO group had a significantly lower pRNFL thickness at 12 and 24 months compared to those of the BRVO group and normal controls.

Glaucoma is a progressive optic neuropathy accompanied by morphological abnormalities of the optic disc and surrounding structures. The measurement of pRNFL thickness is useful to confirm structural changes in early glaucoma^23,24^. Additionally, this technique is superior to topographic optic disc assessment in diagnosing glaucoma^25,26^. Considering the irreversible nature of glaucoma, early detection of pRNFL defects is important for a proper evaluation and for initiating treatment in a timely manner.

In this study, global and all-sector pRNFL thicknesses in the affected eyes in both the BRVO and CRVO groups decreased significantly at 24 months and were significantly greater than those of the fellow eyes at baseline. The pRNFL thickness of the affected eyes at baseline was likely to be greater than that of the fellow eye owing to the structural effect of macular edema. In both groups, the pRNFL thickness in the affected eye decreased as the CMT decreased. In this regard, a previous study including patients with diabetic retinopathy and age-related macular degeneration reported that the pRNFL was affected by macular lesions^27,28^. Macular edema due to RVO is known to involve the inner layers of retina, as compared to diabetic macular edema^29^. Therefore, it is not surprising that the inner RNFL is predominantly affected in RVO.

In contrast, the pRNFL thickness in the superior temporal sector was lower in the affected eyes than in the fellow eyes of the BRVO group at 12 and 24 months. In a study including 20 patients with unilateral BRVO, the mean RNFL thickness was significantly greater in the affected eyes than in the normal fellow eyes at the first visit and at 1 month, while it was lower at 6 and 12 months^18^. However, they reported that the RNFL thickness of the area opposite to that of BRVO did not change significantly at 12 months and showed no difference in comparison with the fellow eyes^18^. This would suggest that the RNFL becomes thin due to BRVO itself. Similarly, in a histopathologic study of patients with BRVO, Frangieh et al.^30^ reported inner retinal ischemic atrophy in the area of occlusion; the decrease in pRNFL thickness of the affected eyes was also considered to be a result of RNFL atrophy. However, in the present study, there was no sector in which the pRNFL thickness of the affected eyes was lower than that of the fellow eyes of the CRVO group at 24 months. Indeed, the pRNFL thickness of the temporal sector remained significantly greater in the affected eye throughout the entire study period. The CMT of affected eyes of the CRVO group was relatively greater than that of the BRVO group, even at 24 months. As a result, the effect of macular edema on pRNFL seemed to persist for up to 24 months in the CRVO group. Follow-up studies are necessary to confirm if this effect persists after macular edema decreases.

RVO has been associated with glaucoma, and the two diseases share common risk factors such as old age, hypertension, diabetes, cardiovascular disease, and hyperlipidemia^11–13,16^. There are also reports of increased blood and plasma viscosity in patients with RVO and glaucoma^31,32^. Pathophysiological changes in blood vessels caused by these risk factors can have a systemic effect. Therefore, if RVO or glaucoma due to vascular abnormality occurs in one eye, similar changes are likely to occur in the fellow eye. In this study, the fellow eyes of patients with BRVO showed no significant differences in pRNFL at any of the visits compared to those of normal controls. However, global pRNFL thickness decreased significantly in the fellow eyes of patients with BRVO, whereas there was no significant decrease in pRNFL in the normal control eyes at 24 months compared to the baseline. In the fellow eyes of patients with CRVO, the global, superior temporal sector, and inferior temporal sector pRNFL thickness decreased significantly at 24 months. These changes in the fellow eyes of RVO support the hypothesis that systemic pathophysiological vascular abnormalities cause both glaucomatous optic neuropathy and RVO. Additionally, Shin et al.^20^ reported that, compared with normal controls, the fellow eyes of patients with unilateral RVO showed a lower peripapillary vessel density and perfusion density in OCT angiography; furthermore, pRNFL thickness was significantly related to peripapillary vessel density and perfusion density. Insufficient blood flow can cause ischemic damage to the optic nerve tissue and axons and may lead to a decrease in pRNF.

In a previous cross-sectional study, the fellow eyes of patients with unilateral RVO had a lower pRNFL thickness compared to normal controls^14,20,21^. Kim et al.^14^ and Shin et al.^20^ reported that the average, inferior quadrant, and temporal quadrant pRNFL thicknesses were significantly lower in the fellow eyes of patients with unilateral RVO (both BRVO and CRVO) than in normal controls. Additionally, Sirakaya et al.^21^ reported that the average and inferior quadrant pRNFL thickness of the fellow eyes of 35 patients with unilateral BRVO was significantly lower than that of healthy controls. Characteristically, a decrease in inferior quadrant pRNFL thickness was observed in all three previously mentioned studies. The inferior quadrant is the area where glaucoma changes are most commonly observed^33^. A previous study investigating longitudinal changes in the pRNFL thickness of fellow eyes of patients with unilateral RVO (both BRVO and CRVO) reported that the annual reduction rate was greater in the fellow eyes compared to those in normal controls^22^. However, the BRVO and CRVO groups were not analyzed separately, and the CRVO group only included eight eyes. Furthermore, patients were not treatment-naïve and the mean pRNFL thickness at baseline seemed to be different between the fellow eyes of patients with RVO (95.9 ± 8.0 μm) and those of normal controls (98.7 ± 7.4 μm). In our study, the global, inferior temporal sector, and inferior nasal sector pRNFL thicknesses at 12 and 24 months were significantly lower in the fellow eyes of patients with CRVO than in both normal controls and fellow eyes of patients with BRVO. Additionally, the reductions in the global, superior temporal sector, inferior temporal sector, and inferior nasal sector pRNFL thicknesses of the fellow eyes of patients with CRVO were greater than those of normal controls and of the fellow eyes of patients with BRVO (Fig. 1). This indicates that the fellow eyes of patients with CRVO are more vulnerable to pRNFL damage.

This study had several limitations, the first of which is its nonrandomized, retrospective design. Second, although we observed a structural change in pRNFL, the result of a visual field test, which measures the corresponding functional change, was not confirmed. Finally, although the prevalence of hypertension and diabetes in the three groups was matched, no data was collected regarding disease severity or specific treatments. It is necessary to compare any differences in blood pressure and laboratory data (such as blood glucose, glycated hemoglobin, lipid tests) between the three groups through follow-up prospective studies. Nevertheless, this study provides important information regarding the differences between branch and central RVO and the association of these conditions with glaucoma.

In conclusion, our study showed that the fellow eyes of patients with BRVO and CRVO suffered a reduction in pRNFL thickness compared with normal controls. Additionally, the fellow eyes of patients with CRVO are susceptible to pRNFL damage during the 2 years after the event. These findings suggest that RVO and glaucoma share common systemic pathophysiological vascular abnormalities. Additionally, our findings show that there might be differences in the pathogenic mechanisms of BRVO and CRVO. This suggests that close observation is needed to evaluate the changes in pRNFL in both the affected and fellow eyes of patients with RVO, especially in those with CRVO.

## Methods

### Ethics statement

This retrospective, longitudinal, case–control study complied with the tenets of the World Medical Association Declaration of Helsinki. The study and data collection were carried out with approval from the Institutional Review Board (IRB) of Hangil Eye Hospital (IRB number: 21010). The requirement to obtain informed consent from study participants was waived by the Hangil Eye Hospital IRB given the retrospective nature of the study.

### Subjects

This study included patients newly diagnosed with unilateral BRVO (BRVO group) or unilateral CRVO (CRVO group) at the Hangil Eye Hospital between April 2015 and June 2018. All patients were diagnosed with RVO using fundus examination, fundus photography, fluorescein angiography, and spectral domain optical coherence tomography (SD-OCT). The normal control group included age-, sex-, and underlying disease (hypertension and diabetes)-matched patients who visited the hospital for an ophthalmic examination within the same period and did not present abnormal findings on fundus examination and SD-OCT.

Patients were excluded if they met any of the following criteria: (1) history of optic nerve disease, such as glaucoma, ischemic optic neuropathy, or optic neuritis; (2) presence of a glaucomatous optic disc (increased cup/disc ratio, narrowing or notching of the neural rim, or disc hemorrhage); (3) history of other retinal disease or uveitis; (4) history of previous treatments that affect RNFL thickness, including intraocular surgery, intravitreal injection, laser photocoagulation, and glaucoma eye drops; (5) spherical equivalent >  ± 6.0 D; or (6) difficulty in obtaining a clear image owing to significant media opacity.

### Ophthalmic examinations

The BCVA and IOP were assessed at baseline and at 6, 12, and 24 months.

**Figure 2:**
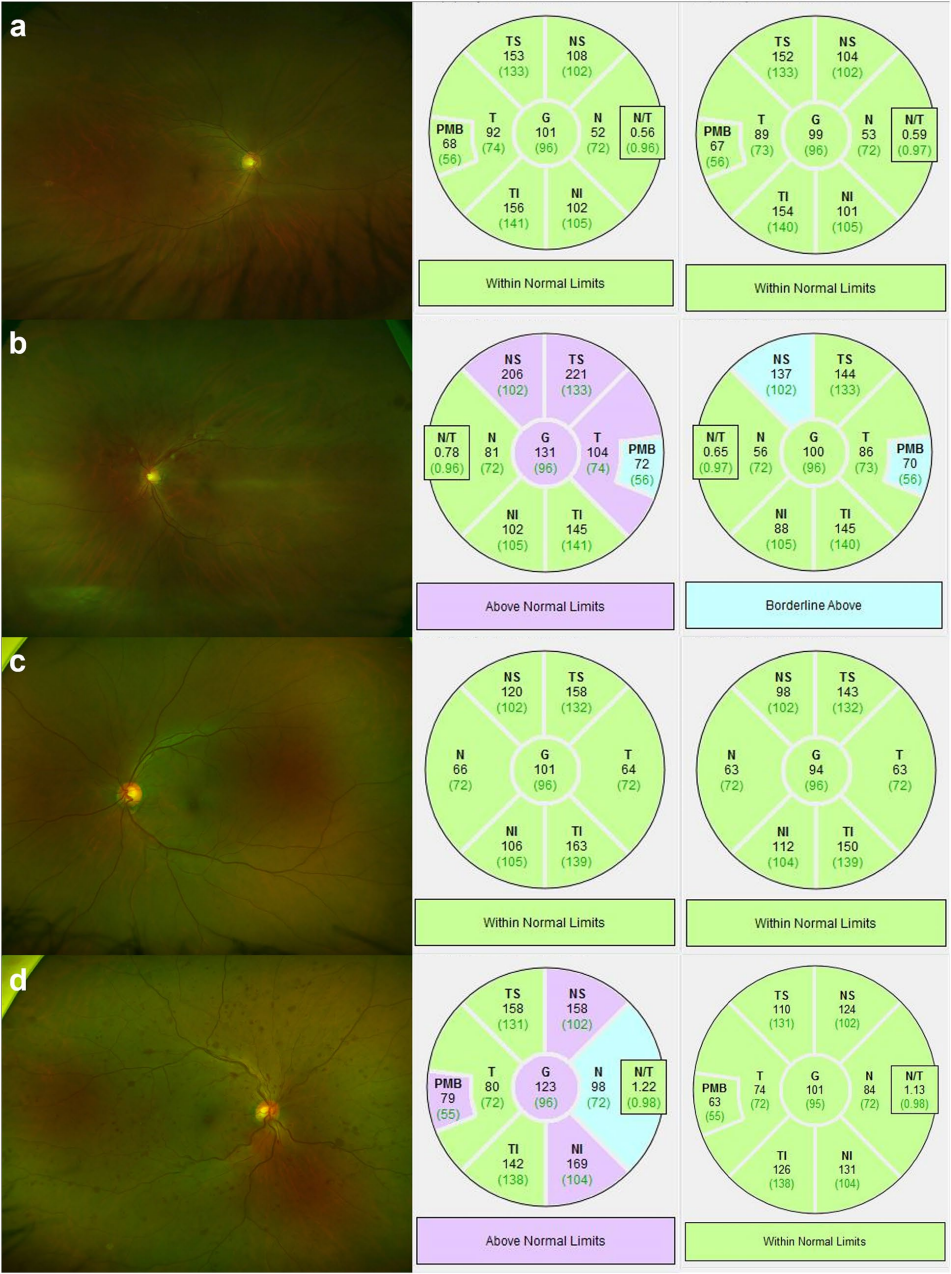
Wide fundus photographs at baseline and peripapillary retinal nerve fiber layer (pRNFL) thickness analysis at baseline and after 24 months. (**a**) The fellow eye of a patient with unilateral branch retinal vein occlusion (BRVO). (**b**) The affected eye of the same patient with BRVO. (**c**) The fellow eye of a patient with unilateral central retinal vein occlusion (CRVO). (**d**) The affected eye of the same patient with CRVO. The global pRNFL thickness of the BRVO fellow eye decreased by 2 μm over 2 years, and that of the CRVO fellow eye decreased by 7 μm. The pRNFL thickness of the affected eye was higher at baseline both in patients with BRVO and CRVO, and the pRNFL thickness of patients with BRVO was characteristically high in the occluded vascular area. *G* global, *TS* temporal superior, *T* temporal, *TI* temporal inferior, *NI* nasal inferior, *N* nasal, *NS* nasal superior.

The IOP was measured with a noncontact tonometer (NT-530, NIDEK, Aichi, Japan); the average value was measured three times for each eye and was displayed on the machine. pRNFL thickness and CMT were measured using SD-OCT (Spectralis^®^ OCT, Heidelberg Engineering, Heidelberg, Germany) and the Spectralis Nsite Axonal Analytics Module (Acquisition software version 6.12.4.0) (Heidelberg Engineering). pRNFL thickness was automatically measured at six sectors (Fig. 2): temporal (315–45°), superior temporal (45–90°), superior nasal (90–135°), nasal (135–225°), inferior nasal (225–270°), and inferior temporal (270–315°). Global RNFL thickness was obtained by averaging 360° pRNFL thickness measurements.

### Statistical analysis

Statistical analyses were performed using the Statistical Package for the Social Sciences software version 23.0 (IBM, Armonk, NY, USA). Results are expressed as mean ± standard deviation. One-way analysis of variance (ANOVA) or Pearson’s chi-square test were performed to compare the demographics and characteristics among the three groups. Comparisons between the affected and fellow eyes were conducted using paired *t*-tests. Changes in values between baseline and 24-month follow-up examinations were analyzed using a paired *t-*test. An independent samples *t-*test was used to compare pRNFL thickness between the fellow eyes of patients with unilateral RVO and the eyes of normal controls. A repeated-measures ANOVA was used to compare the differences between groups regarding pRNFL changes over time. Statistical significance was set at p < 0.05.

## Data Availability

The datasets used and/or analyzed during the current study are available from the corresponding author on reasonable request.
